# Effects of kaolinite layer expansion and impurities on the solid-state reaction of kaolinite†

**DOI:** 10.1039/d1ra07762g

**Published:** 2021-11-30

**Authors:** Shingo Machida, Ken-ichi Katsumata, Atsuo Yasumori

**Affiliations:** Department of Material Science and Technology, Faculty of Advanced Engineering, Tokyo University of Science 6-3-1 Niijuku, Katsushika-ku Tokyo 125-8585 Japan shingo.machida@rs.tus.ac.jp

## Abstract

Expanded kaolinite without impurities was found to serve as a suitable raw material for the rapid formation of metastable CaAl_2_Si_2_O_8_ with the suppression of byproduct formation. This was accomplished based on the solid-state reaction of the kaolinite with calcium carbonate promoted by mechanical grinding.

## Abstract 2

Effects of kaolinite layer expansion and impurities on the rapid formation of metastable CaAl_2_Si_2_O_8_ with the supression of byproduct formation were demonstrated based on the solid-state reaction of calcium carbonate with methoxy-modified kaolinite.

## Graphical abstract

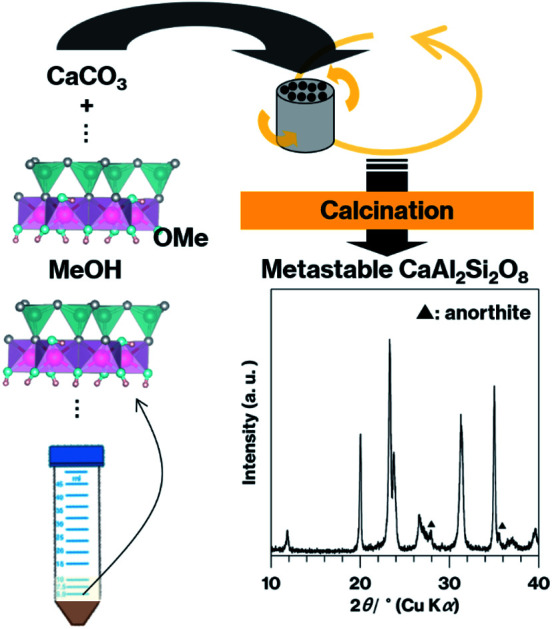

## Introduction

Various inorganic solids can be formed *via* solid-state reactions induced by grinding two or more raw materials together to activate and increase their surface area.^1–3^ These grinding effects are especially advantageous in the case of layered inorganic solids^4^ since these materials comprise stacked layers whose stacking order and structure can be disrupted by grinding.^5–9^ The degree of disruption can be increased as these inorganic layers expand upon intercalation, a process in which guest species are inserted between stacked layers to form intercalation compounds comprising alternately stacked inorganic and guest species layers.^4^ Therefore, intercalation compounds may have applications in solid-state reactions in which the absence of impurities is desirable.

Herein, we report the effects of the expansion of kaolinite layers and of the presence of impurities on the solid-state reaction of this material. Kaolinite, a layered aluminosilicate having the formula Al_2_Si_2_O_5_(OH)_4_, is well-known as a raw material for the synthesis of various inorganic solids.^10–13^ Each neutral and asymmetrical layer within kaolinite comprises an AlO_2_(OH)_4_ sheet and a SiO_4_ sheet. These sheets form hydrogen bonds at kaolinite interlayers where neither ions nor molecules are originally present. Additionally, kaolinite undergoes intercalation of salts and neutral molecules bearing polar groups. These groups break/weaken hydrogen bonds between AlO_2_(OH)_4_ and SiO_4_ sheets at pristine kaolinite interlayers, to expand kaolinite layers and to form intercalation compounds.^14–32^ These compounds easily undergo the disruption of the stacking order by grinding compared to pristine kaolinite.^7,9^ Among compounds prepared using kaolinite intercalation chemistry, methoxy-modified kaolinite (MeO-Kaol) is organically-modified kaolinite with the formula Al_2_Si_2_O_5_(OH)_4−*x*_(OCH_3_)_*x*_ (in which *x* never exceeds 1)^16,17^ and can accommodate water molecules under ambient conditions in a stable manner. In this scenario, the basal spacing of the material is expanded from 0.72 nm of pristine kaolinite to 0.86 nm.^16,17^ MeO-Kaol is also able to intercalate methanol molecules when immersed in this solvent, to increase the basal spacing to 1.12 nm.^17^ Interestingly, white and brown regions of the kaolinite are distinguishable following the methoxylation process,^17^ with the latter color likely due to the presence of impurities. The study reported herein assessed the solid-state reaction of MeO-Kaol with calcium carbonate (CaCO_3_), a low-cost material, to form a metastable CaAl_2_Si_2_O_8_ phase. Metastable CaAl_2_Si_2_O_8_ containing anorthite, the stable phase of CaAl_2_Si_2_O_8_, can be produced by the calcination of a mixture of ground CaCO_3_ and Georgia kaolinite (GK), a brown kaolinite, for 12 h.^10^ Metastable CaAl_2_Si_2_O_8_, a series of layered inorganic solids having the formula RAl_2_Si_2_O_8_ (where R is an alkali metal cation)^33^, exhibits phosphorescence when doped with rare earth ions.^34^ Additionally, the CaO–Al_2_O_3_–SiO_2_ glass–ceramic precipitated metastable CaAl_2_Si_2_O_8_ crystals shows improved mechanical properties and unique fracture behavior^35^ that is currently under investigaiton. Amorphous metastable CaAl_2_Si_2_O_8_ has also been shown to efficiently remove heavy metal ions from solutions in conjunction with a minimal increase in pH.^11^ Based on these applications, it would be helpful to develop a means of efficiently synthesizing this material. The present research initially examined the effects of kaolinite layer expansion using GK, a well-crystallized kaolinite that displays significant intercalation capabilities.^19^ The effects of impurities were subsequently assessed in trials with Kanpaku kaolinite (KP), a white kaolinite, during which this material was treated with acid and impurities were removed during methoxylation.^17^ These different kaolinites were ground with CaCO_3_ then subjected to calcination to form metastable CaAl_2_Si_2_O_8_. The calcination mechanism was investigated by trials using halloysite, a tubular aluminosilicate with the same formula as kaolinite, as a raw material. It should be noted that, in the halloysite structure, the AlO_2_(OH)_4_ and SiO_4_ surfaces appear at interiors and exteriors of the tubes respectively.^36^

## Experimental

In these trials, 5 g portions of GK (KGa-1b, obtained from the Source Clays Repository of the Clay Material Society of the U.S.A.) or KP (JSCC-1101c, obtained from the Clay Science Society of Japan) were soaked in 50 mL of 1 M hydrochloric acid for three days to produce the specimens referred to as A-KP and A-GK, respectively. The methoxy modifications of GK, KP and A-KP were conducted according to a previously reported method^17,18^ to give the products designed herein as Me-GK, Me-KP and Me-A-KP. During these preparations, the upper and lower regions of centrifuged products, which exhibited different colorations, were separated in a manner similar to that in a previous study.^18^ These upper and lower regions are denoted herein as U-X and L-X, where X represents the type of kaolinite and the products. Following these procedures, KP and Me-KP were dispersed in methanol and allowed to stand for a day. A sample of methanol-dispersed KP also underwent immediate centrifugation.

A quantity of the kaolinites or halloysite (obtained from Sigma-Aldrich Co. Ltd.) was mixed with CaCO_3_ (obtained from Hayashi Pure Chemical Int., Ltd.) in a 1 : 1 molar ratio using an agate mortar and pestle to generate the specimens referred to herein as the ungrounded raw materials. A 720 mg quantity of each mixture were subsequently dispersed in 8 mL of methanol and wet milled in a planetary ball mill at 250 rpm for 12 h using a resin vessel (12.5 mL) and 120 silicon carbide balls (3 mm in diameter). After milling, the solids were centrifuged at 4000 rpm for 1 min and then dried at 80 °C for 1 h to provide the specimens referred to herein as the ground raw materials. After drying, portions of these products were calcined at 900 °C for 1.5, 3, or 4.5 h to prepare the products denoted as Y + C-1.5, 3, or 4.5 h, where Y and C represent raw materials and CaCO_3_, respectively.

X-ray diffraction (XRD) patterns (XRD-6100, Shimadzu) were acquired from kaolinite and the products to assess the stacking order and crystalline phases. Fourier-transform infrared (IR) spectra (FT/IR-4100, JASCO) were obtained by KBr method to investigate the degree of structural breakdown of the products. Field-emission scanning electron microscopy (FE-SEM; Supra40 microscope, Zeiss) was used to characterize particle sizes and morphologies.

## Results and discussion

**Fig. 1:**
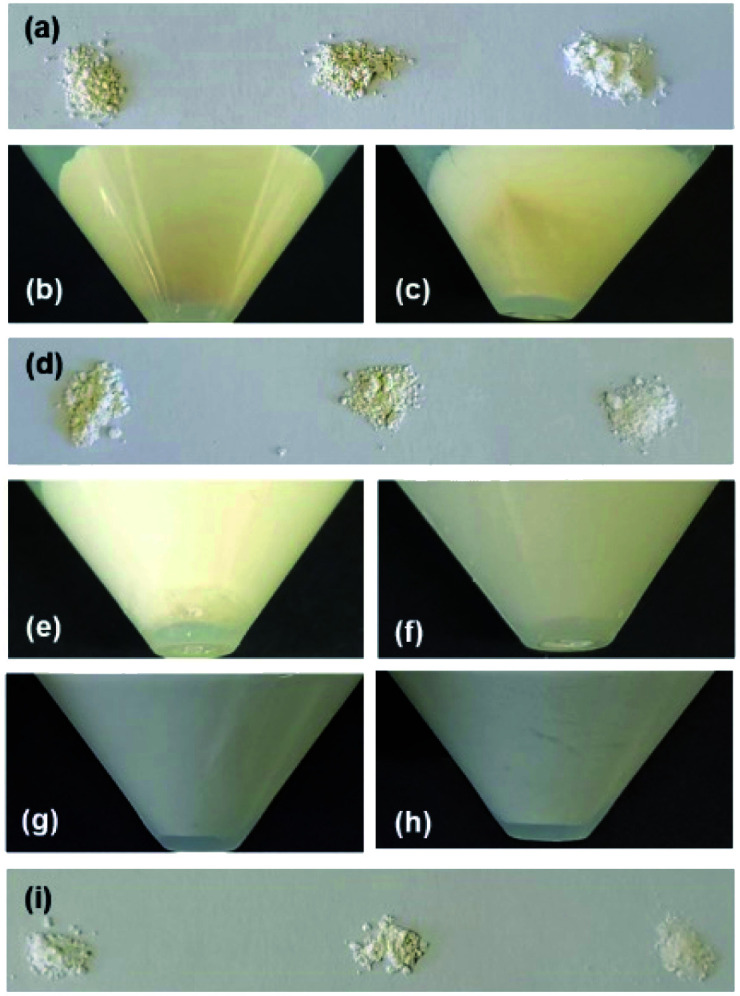
Photographs of (a) GK (left), A-GK (middle) and KP (right), (b) Me-GK after centrifugation during preparation process, (c) Me-KP after centrifugation, (d) U-Me-GK (left), L-Me-GK (middle) and U-Me-KP (right), (e) Me-A-KP after centrifugation, (f) methanol-dispersed Me-KP after standing for one day, (g) methanol-dispersed KP after standing, (h) centrifuged KP after being dispersed in methanol, and (i) U-Me-A-KP + C-4.5 h (left), Me-GK + C-4.5 h (middle) and KP + C-4.5 h (right).

The brown color of GK changed only minimally following the acid treatment to produce A-GK, while the original KP was white (Fig. 1a); thus, the methoxylation of A-GK was not conducted (see Experimental). Brown and relatively white regions were observed in the products after methoxy modification of GK and KP (Fig. 1b and c). Both U-Me-GK and L-Me-GK were brown, whereas U-Me-KP was white (Fig. 1d). Additionally, the proportion of the brown region decreased after methoxy modification of A-KP (Fig. 1e). A brown region did not appear when methanol-dispersed KP and Me-KP were allowed to stand for a day, and after which the former was immediately centrifuged (Fig. 1f–h). U-Me-A-KP + C-4.5 h was white, while Me-GK + C-4.5 h and KP + C-4.5 h appeared light yellow and pink, respectively (Fig. 1i).

It is well known that kaolinite can undergo incomplete intercalation,^9^ which is demonstrated by the continued presence of an XRD peak corresponding to a *d* value of 0.72 nm (due to the original kaolinite layers) following intercalation. The ratio of the intensity of the diffraction line due to expanded kaolinite layers to that of the line due to the original kaolinite layers can therefore be used as an indicator of the extent of intercalation.^20–23^ In general, GK generally exhibits greater intercalation capability than KP.^19^

**Fig. 2:**
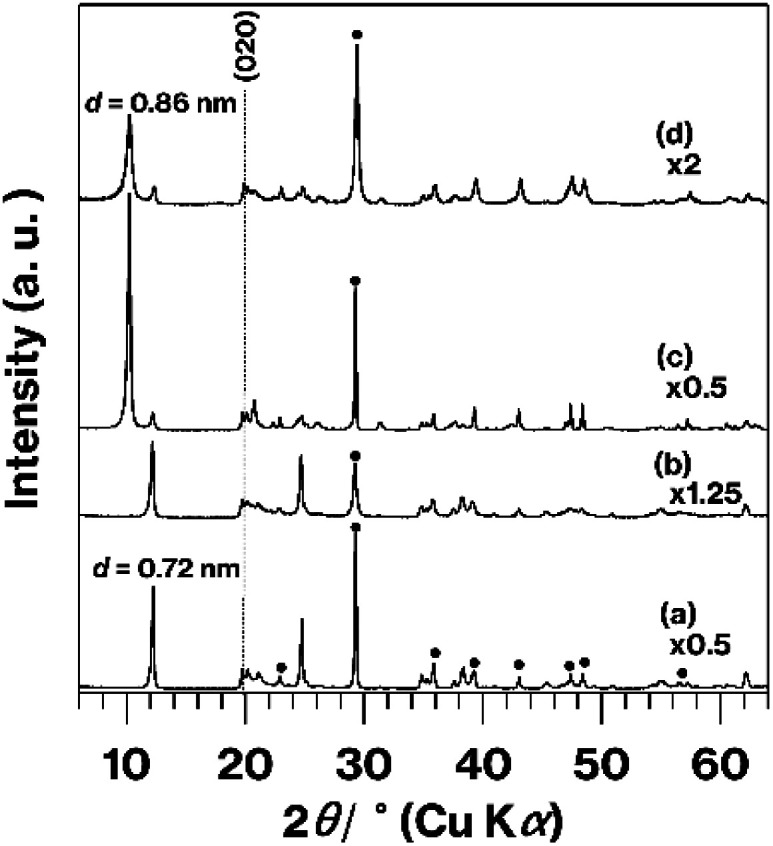
XRD patterns for GK mixed with CaCO_3_ (a) before and (b) after grinding, and for Me-GK mixed with CaCO_3_ (c) before and (d) after grinding. Reflections due to CaCO_3_ are indicated by filled circles in (a).

Fig. 2 presents XRD patterns for ground and unground GK mixed with CaCO_3_. After grinding, the ratio of the intensity of the diffraction line (*d* = 0.86 nm) due to the basal spacing of Me-GK to that of the (020) diffraction line due to the lateral atomic arrangement of the kaolinite layers decreased relative to the ratio of the intensity of the diffraction line (*d* = 0.72 nm) due to the basal spacing of pristine kaolinite to this same (020) line.^24^ In order to easily understand the degree of stacking order in each raw material, we tentatively show the relative intensities of reflection due to (001), basal spacings, with respect to those due to (020) (*I*_(001)_/*I*_(020)_); 4.8 for GK mixed with CaCO_3_ before grinding, 3.8 for that after grinding, 11 for Me-GK mixed with CaCO_3_, and 4.2 for that after grinding. Thus, the stacking order in MeO-GK was greatly perturbed compared with that in pristine kaolinite. The relative intensities of the reflections ascribed to CaCO_3_ also decrease after grinding.

Fig. S1† shows IR spectra in the OH stretching region of GK, Me-GK, and GK-based ground raw materials. The kaolinite spectrum (Fig. S1a†) generally exhibits three OH stretching bands at 3696, 3670, and 3653 cm^−1^ due to inter-layer hydroxyl groups and one OH stretching band due to inner-layer hydroxyl group. The former three bands are perturbed by intercalation of guest species, while the latter cannot undergo such perturbation. When the intercalation reaction proceeds, the intensities of the three former bands relative to the later band decrease compared to those obtained from the kaolinite spectrum.^14,37,38^ Additional OH stretching bands/shoulders due to interlayer hydroxyl groups hydrogen-bonded to the guest species can appear at lower wavenumbers.^17,37,38^ In this study, the MeO-Kaol spectrum (Fig. S1b†) fits well with that reported previously.^17^ Meanwhile, four OH stretching bands are broaden when kaolinite undergoes mechanical grinding due to the structural breakdown.^7^ In the present study, the profiles of ground raw materials are slightly broadened compared to GK and Me-GK (Fig. S1c and d†). The degrees are lesser extent than those reported in previous studies.^7,39^ Compared to GK, Me-GK exhibits the more spectrum broadening after grinding (Fig. S1c and d†).

**Fig. 3:**
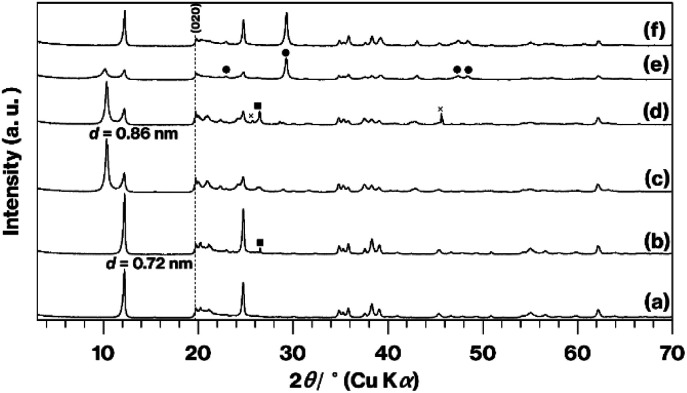
XRD patterns for (a) KP, (b) A-KP, (c) U-Me-A-KP, (d) L-Me-A-KP, (e) ground U-Me-A-KP mixed with CaCO_3_, and (f) ground KP mixed with CaCO_3_. Reflections due to CaCO_3_ are indicated by filled circles and squares, while the cross mark represents an unknown phase.

Fig. 3 shows XRD patterns obtained from the original KP, the KP-based products, and the raw materials after grinding with CaCO_3_. There are no significant differences in the patterns generated by KP and A-KP (Fig. 3a and b), although the latter includes a weak reflection resulting from quartz^25^ (Fig. 3b). This quartz reflection also appears in the XRD pattern for L-Me-A-AP (Fig. 3d) but not in that for U-Me-A-AP (Fig. 3c). The reflections due to impurities^40^ acquired from U-Me-KP and L-Me-KP (Fig. S2†) were less intense in the pattern obtained from U-Me-A-KP (Fig. 3c). After grinding, the profile changes for KP or U-Me-A-KP mixed with CaCO_3_ by grinding (Fig. 3e and f) are similar to those for GK or Me-GK mixed with CaCO_3_ by grinding as shown in Fig. 2c and d.

**Fig. 4:**
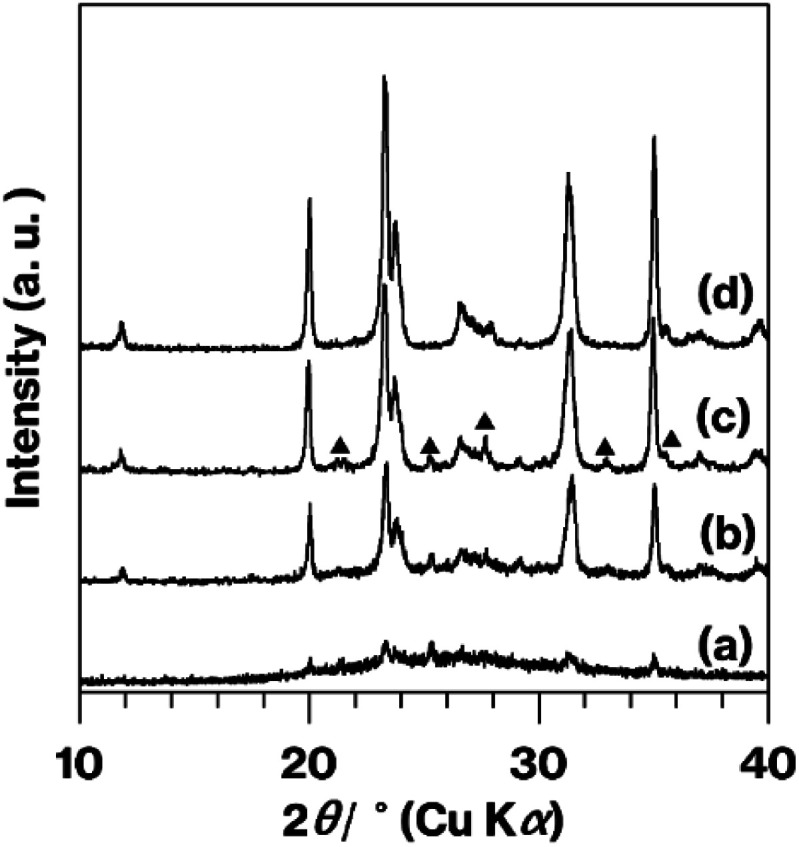
XRD patterns for (a) GK + C-3 h, (b) Me-GK + C-3 h, (c) Me-GK + C-4.5 h, and (d) U-Me-A-KP + C-4.5 h. Reflections due to anorthite are indicated by filled triangles in (c).

Fig. 4 provides XRD patterns for the calcined products. Compared with GK + C-3 h, which essentially generated a halo XRD pattern (Fig. 4a), Me-GK + C-3 h (Fig. 4b) provided a pattern that agrees with that reported for metastable CaAl_2_Si_2_O_8_.^10^ Additionally, there are some weak diffraction lines due to anorthite^10^ in this pattern (Fig. 4b). Compared with the Me-GK + C-3 h pattern (Fig. 4b), the intensities of the reflections resulting from metastable CaAl_2_Si_2_O_8_ and anorthite are greater in the Me-GK + C-4.5 h pattern (Fig. 4c). In addition, relative to the Me-GK + C-4.5 h pattern (Fig. 4c), the intensities of the reflections due to metastable CaAl_2_Si_2_O_8_ are slightly increased while those of the anorthite reflections are decreased for U-Me-A-KP + C-4.5 h (Fig. 4d).

The patterns obtained from Me-GK + C-3 h, Me-GK + C-4.5 h and U-Me-A-KP + C-4.5 h (Fig. 4b–d) are similar to those reported previously, which indicates the formation of metastable CaAl_2_Si_2_O_8_ and a small amount of anorthite after a 12 h calcination at 900 °C following the planetary milling of a mixture of GK and CaCO_3_ for 12 h.^10^ The U-Me-A-KP pattern in particular displays weaker anorthite reflections. Note that the ground GK and CaCO_3_ sample that was calcined at 800 °C for 12 h generated a halo XRD pattern as a consequence of the decomposition of the CaCO_3_ and dehydroxylation of the kaolinite to produce metakaolinite, an amorphous layered aluminosilicate.^26,41^

Prior study determined that the grinding induced the breakdown of both Al–O–Si and Si–O–Si bonds to form new hydroxyl groups with adsorbed water, based on the mass loss due to H_2_O release estimated using thermogravimetry (TG) analysis.^10^ It is also known that a portion of the hydroxyl groups in MeO-Kaol is replaced with methoxy groups.^16,17^ The TG curve obtained from the MeO-Kaol shows overlapping mass losses due to kaolinite dehydroxylation and degradation of methoxy groups.^16,17,25^ CHN analysis is therefore required to determine the carbon content due to methyl groups.^16,17,25^ However, the amount of methoxy groups in the ground raw materials cannot be detected since they contain CaCO_3_ which releases carbons due to the decarbonation by heating. Additionally, the amounts of protons in methoxy groups cannot be also detected since those in water due to the dehydroxylation of kaolinite are overlapped. Therefore, the present study cannot clarify the mass loss related to the removal of H_2_O from the material. Although kaolinite hydrates can be prepared without methoxylation^27^ and could be used as a raw material for wet grinding in water, kaolinite hydrates are often unstable under ambient conditions as a result of the release of interlayer water, which can destroy the stacking order.^28^ That is, deintercalation of water molecules can lead to the appearance of disordered stacking. The release of guest species can shift the dehydroxylation of kaolinite to lower temperatures,^9^ and so it is difficult to assess the true mass loss associated with the release of H_2_O generating from the breaking of kaolinite bonds such as Al–O–Si bonds. Concerning the breakdown of kaolinite structure by grinding, the effect is lesser extent compared to those reported in previous studies^7,39^ based on IR spectra (Fig. S1†). This permits us to focus the difference in the staking order disrupted by grinding between expanded and pristine kaolinites.

The solid-state reaction of kaolinite involves metakaolinite.^3^ Although metakaolinite generally does not produce XRD diffraction lines because it has an amorphous layered structure, we recently succeeded in the formation of orderly stacked metakaolinite and revealed the destruction of the ordered structure upon manual grinding.^26^ Thus, before grinding and calcination of such raw materials, it would be beneficial to assess the effects of pre-heating kaolinite to form metakaolinite with various stacking orders. Additionally, the reaction of CaCO_3_ with metakaolinite is worth further investigation. Our group therefore intends to continue our study of such materials. In this scenario, the use of organic solvents as media for wet grinding could be helpful to suppress the rehydration of metakaolinite.^42^

Based on the appearance of the various products (Fig. 1) and the XRD patterns (Fig. 3), the presence of impurities in the kaolinite was eliminated by combining the acid treatment, methoxy modification and centrifugation when KP was used. The separation of the white and brown regions by centrifugation was not possible without methoxylation and natural sedimentation, although the details of the associated mechanisms are not yet clear. The XRD patterns for the calcined products (Fig. 4) showed a lack of impurities when U-Me-A-KP was employed as raw materials for the rapid formation of metastable CaAl_2_Si_2_O_8_, with suppression of the anorthite phase and a product that was truly white (Fig. 1i). In general, the brown color of aluminosilicate clay minerals is due to impurities and small amounts of Fe substituted for Al or Mg. This substituted Fe likely remains in the calcined products, based on the results for Me-GK and KP (Fig. 1i). In the case of the GK, however, the more stronger acid treatment may decompose the kaolinite structure. The data also indicate that quartz in the KP could be removed by the present procedures (Fig. 3 and S2†). Additionally, based on the reduced structural and morphological decompositions (Fig. S3 and S4†) of halloysite, in which the tubular exteriors have SiO_4_ surfaces, the reaction of CaCO_3_ with a silica-like surface appears to promote the formation of an anorthite phase. The impurities in synthetic kaolinite comprise small amounts of boehmite and gibbsite,^43,44^ although the intercalation capability of this material has been examined solely using dimethylsulfoxide^21^ as an effective molecule for direct intercalation between kaolinite layers.^15,16,20–22^ Thus, when expanded synthetic kaolinites are used as raw materials in solid-state reactions, the intercalation capacity of each material should be investigated in detail using various polar molecules, as conducted in previous studies using GK and KP.^15–21,23–28^ Additionally, a centrifugation to separate impurities as conducted in the present study should be examined. Therefore, the use of other kaolinites could require further basic studies.

The present XRD patterns (Fig. 2 and 4) and previous results^10^ (see two paragraphs before) reveal the rapid formation of metastable CaAl_2_Si_2_O_8_ when using expanded kaolinite as a raw material. It is well known that methanol molecules cannot intercalated between kaolinite layers.^27^ Thus, the expansion of the kaolinite layers upon methanol accommodation facilitated the destruction of the stacking order of the kaolinite layers when MeO-Kaol underwent wet grinding in methanol. The degree of stacking disorder observed in this study was also greater than that reported in a previous study that assessed the wet grinding of GK and CaCO_3_ in water using a planetary ball mill.^10^

When the calcined product was prepared using halloysite as the raw material, the XRD pattern for halloysite + C-4.5 h (Fig. S3a†) included reflections due to metastable CaAl_2_Si_2_O_8_, along with anorthite reflections that were more intense compared with the XRD patterns shown in Fig. 4. There were no significant differences in the patterns (Fig. S3b and c†) and the tube morphology (Fig. S4†) of the halloysite before and after grinding.

Since kaolinite occurs naturally, it will unavoidably have variable levels of crystallinity. Effect of crystallinity on the present results as well as kaolinite intercalation capability are thus one of interests, while particle size distributions and impurities are also different for each kaolinite produced in nature. Note that the kaolinite intercalation capability depends on the lateral sizes of kaolinite platy particles.^14^ Additionally, to the best of our knowledge, even in the case of synthetic kaolinite, the size and distributions are not well controlled. Meanwhile, methoxy-modified kaolinite has been used as a versatile intermediate to intercalate various organic molecules and polymers^15–18,24–26^ and expansion of the kaolinite layers up to a spacing of approximately 4 nm has been reported.^24^ This level of expansion could also be sufficient to destroy the stacking order of the kaolinite layers during grinding. Methoxy-modified kaolinite can also be prepared by intercalation of kaolinite with urea,^29^ which is as inexpensive as methanol. Therefore, the present results demonstrate the potential industrial uses of kaolinite.

## Conclusions

In summary, we have demonstrated the effects of kaolinite layer expansion and of the presence of impurities on the rapid formation of metastable CaAl_2_Si_2_O_8_ with suppressed byproduct formation *via* a solid-state reaction of kaolinite, especially MeO-Kaol, with CaCO_3_. The present method can be applied to kaolinite, other layered inorganic solids and/or their intercalation compounds ground with various raw materials such as carbonates.^45–48^

## Author contributions

Shingo Machida: conceptualization, data curation, investigation, project administration, writing-original draft, supervision. Ken-ichi Katsumata: project administration. Atsuo Yasumori: project administration.

## Conflicts of interest

There are no conflicts to declare.

## Supplementary Material

RA-011-D1RA07762G-s001
