# Supplementary material for: Effects of kaolinite layer expansion and impurities on the solid-state reaction of kaolinite
Source: RSC Adv. 2021 Nov 30;11(61):38473–7. doi: 10.1039/d1ra07762g (PMC9043916; doi:10.1039/d1ra07762g)
Supplement: RA-011-D1RA07762G-s001 [file RA-011-D1RA07762G-s001.pdf]

Electronic Supplementary Material (ESI) for RSC Advances.
This journal is © The Royal Society of Chemistry 2021

Electrical Supporting Information

Effects of kaolinite layer expansion and impurities on the solid-state reaction of kaolinite

Shingo Machida, Ken-ichi Katsumata, and Atsuo Yasumori*

Department of Material Science and Technology, Faculty of Advanced Engineering, Tokyo University of Science,

6-3-1 Niijuku, Katsushika-ku, Tokyo 125-8585, Japan

*E-mail: shingo.machida@rs.tus.ac.jp

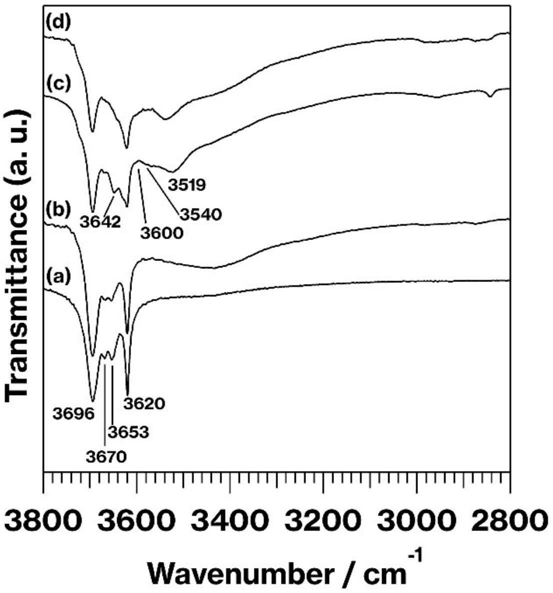

Figure S1. IR spectra in the OH stretching region for (a) GK, (b) GK mixed with $CaCO_3$ after grinding, (c) Me-GK, and (d) Me-GK mixed with $CaCO_3$ grinding.

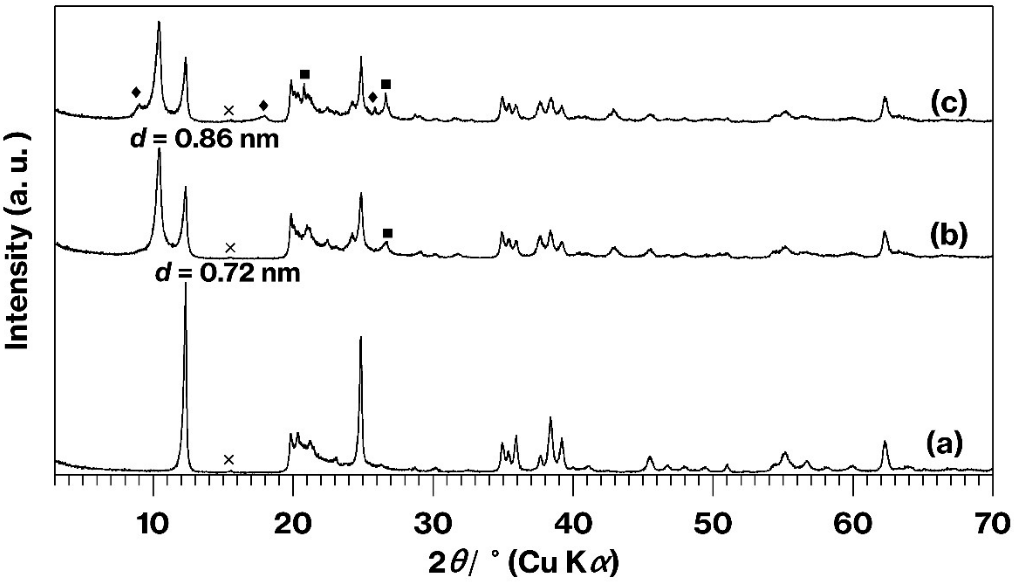

Figure S2. XRD patterns of (a) KP, (b) U-Me-KP, and (c) L-Me-KP. Reflections due to quartz and illite are marked as filled squire and diamond, and a cross mark represents an unknown phase.

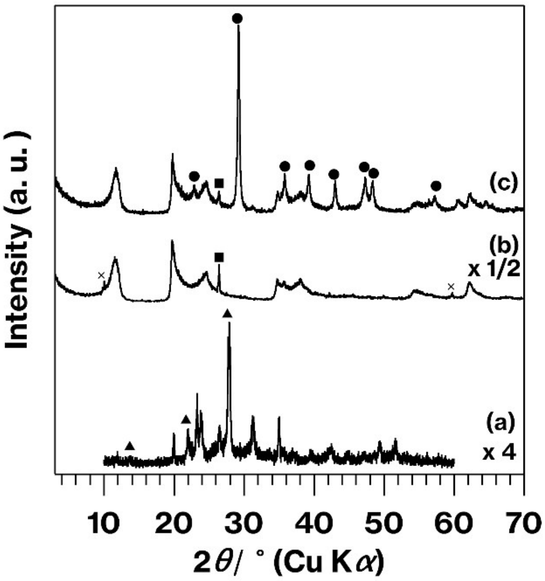

Figure S3. XRD patterns for (a) halloysite+C-4.5 h, (b) halloysite, and (c) ground halloysite mixed with $CaCO_3$. Reflections due to anorthite, $CaCO_3$ and quartz are indicated by filled triangles, circles and squares, respectively, while the cross mark indicates an unknown phase.

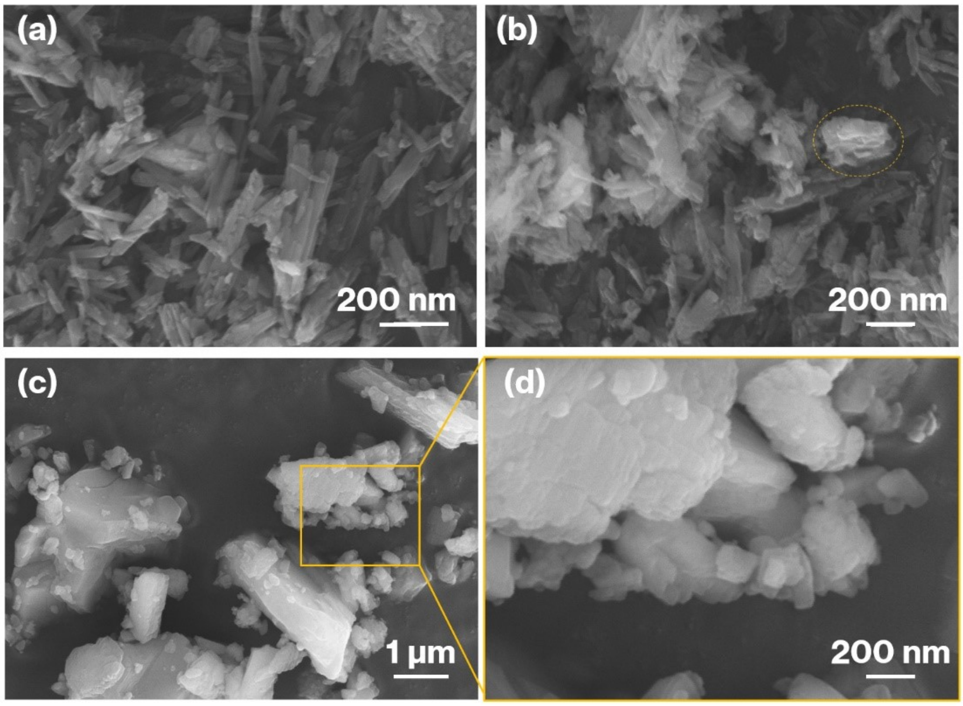

Figure S4. FE-SEM images of (a) halloysite, (b) ground halloysite mixed with $CaCO_3$ (dashed ellipse indicates a representative $CaCO_3$ particle), and (c) $CaCO_3$, and (d) magnified view of region in orange rectangle shown in (c).
